# Pathological and Cytological Studies on Hepatocellular Carcinoma in Cattle Slaughtered at Bishoftu Elfora Abattoir, Central Ethiopia

**DOI:** 10.1155/2021/6649172

**Published:** 2021-05-05

**Authors:** Mesfin Mathewos

**Affiliations:** School of Veterinary Medicine, Wolaita Sodo University, P. O. Box 138, Wolaita Sodo, Ethiopia

## Abstract

Hepatocellular carcinoma (HCC) is one of the most common neoplasms that has been described in many domestic animal species. Hence, the disease has significant economic importance; thus, this study aimed to describe the cytopathological characteristics of hepatocellular carcinomas in cattle slaughtered at Bishoftu Elfora Abattoir, Central Ethiopia. A cross-sectional study design with a purposive sampling technique was performed from October 2017 to May 2018 using macroscopic, histopathologic, and cytological methods. For that matter, a total of sixty cattle were assessed for the presence of a hepatic tumor; however, only 1/60 (0.6%) case was found to be affected by hepatocellular carcinomas. On gross examination, hepatocellular carcinomas exhibited soft, white, multifocal nodules (10–40 mm in diameter) on different lobes of the liver. On the cut surface, the tumor revealed a sharply circumscribed border and was divided into lobules by thin connective tissue. The central zone of the tumors exhibited depression with a whitish fibrous area. Moreover, on histopathology, the tumors divulged unencapsulated carcinomatous lesions consisting of a thick, compact, somewhat ambiguous trabecular pattern of arrangement that was unglued by thin collagenous stroma. Cytological studies suggest that the tumor cells showed anisocytosis, anisokaryosis, prominent nucleoli, multinuclearity, palisading arrangements of neoplastic cells, increased N : C ratios, light eosinophilic cytoplasm, high mitotic index, and cytoplasmic and intranuclear vacuoles. In conclusion, cytopathological findings support a diagnosis of HCC in the liver; thus, further studies with a large sample size and use of immunohistochemistry are important for further characterization of hepatocellular carcinomas in cattle.

## 1. Background

Hepatocellular carcinoma (HCC) is one of the most common neoplasms in the world and has been described in many domestic animal species including cattle, sheep, pigs, cats, dogs, potbellied pigs, and horses [1–3]. Surveys from abattoirs in the United Kingdom indicated that hepatocellular neoplasms are 4 times more common in cattle than sheep and nearly 18 times more common in cattle than pigs [3, 4]. However, dogs may have a higher incidence of hepatocellular carcinomas than most other species, based on several studies, but such reports may simply reflect a disproportionate interest in neoplasms of dogs [5, 6]. Hepatic neoplasms account for 10% of all neoplasms in cattle, 31% in sheep, and 4% in pigs [7]. There is no clear evidence of a gender‐related incidence, and no breed predisposition has been identified [8]. In human medicine, HCC is reportedly the sixth most prevalent cancer and the third most frequent cause of cancer-related death [9, 10].

The etiology of the spontaneously occurring hepatocellular carcinoma in domestic animals is unknown [11–13], but chronic infections (parasites, viruses, and bacteria), hereditary, or chemical ingestion (aflatoxins, pyrrolizidines, and nitrosamines) may play a role in tumor development [3, 14].

Hepatocellular carcinoma can be found in all lobes of the liver. Grossly, HCC has a range of appearances including massive, nodular, or diffuse forms [3]. Of these, massive is the most common, at least in cattle and dogs, and the diffuse pattern is rare [5, 6]. Massive hepatocellular carcinomas are usually a single neoplasm that involves one or contiguous liver lobes. Cut surfaces often reveal multiple smaller nodules within the larger mass that have slightly different appearances [15]. Nodular hepatocellular carcinomas form scattered nodules, often within multiple liver lobes. They vary from small, round, discrete lesions which are a few centimeters in diameter to large, diffuse masses that may be greater than 10 cm in diameter [16]. Diffuse hepatocellular carcinomas are characterized by minute indistinct masses spread throughout the liver parenchyma, affecting multiple lobes. The tumor is paler than normal liver parenchyma, and in well-differentiated cases, it may have a greenish hue as a result of bile accumulation [17].

Microscopically, tumors range from well differentiated to highly anaplastic. Based on the structural organization, HCC has four histologic classifications including trabecular, pseudo-glandular, compact, and scirrhous. Among them, the trabecular pattern is the most common. The pseudo-glandular pattern has malignant hepatocytes surrounding a lumen that may contain bile, with some of these tumors having clear cells because of glycogen or fat. Scirrhous, the least common pattern, contains fibrous stroma separating the tumor cell plates [3, 17].

The cellular features of hepatocytes in hepatocellular carcinomas can be varied. Hepatocytes in well-differentiated carcinomas have central, round nuclei and normally abundant and moderately eosinophilic cytoplasm, similar to normal hepatocytes. The cytoplasm, however, may be pale staining or even vacuolated if filled with glycogen or lipid. However, the poorly differentiated hepatocellular carcinomas are characterized by the presence of pleomorphic hepatocytes with nuclear or cytoplasmic variations, tumor giant cells, eosinophilic to light basophilic cytoplasm with an increased nuclear‐to‐cytoplasmic ratio, round or, rarely, spindle shape cells, enlarged nucleoli, individual hepatocyte giant cells with enlarged or multilobulated nuclei or multinucleated giant cells, mitotic figures, and vascular invasion [1].

The diagnosis of hepatocellular carcinoma has relied on a combination of gross examination too with microscopic features of lesions. The historical failure to distinguish benign from malignant hepatocellular neoplasms in many reports creates a significant challenge in estimating tumor incidence. Up-to-date characterization using contemporary diagnostic criteria is needed to establish the characteristics of hepatocellular carcinomas in cattle [3]. Hence, it would be useful to characterize hepatocellular carcinomas through cytological and histopathological diagnostic tools. Therefore, this study aimed to determine the cytopathological features of hepatocellular carcinomas in cattle.

## 2. Materials and Methods

### 2.1. Study Areas

The study was conducted from October 2017 to May 2018 to evaluate cytopathological characteristics of hepatocellular carcinomas in cattle slaughtered at Bishoftu Elfora Abattoir. Bishoftu is located 45 km southeast of Addis Ababa. The area is located at 9°N latitude and 40°E longitude at an altitude of 1850 meters above sea level (masl) with an annual rainfall of 866 mm of which 84% is in the long rainy season (June to September). The dry season extends from October to February. The mean annual maximum and minimum temperatures are 26°C and 4°C, respectively, with a mean relative humidity of 61.3% [18]. The domestic animals raised in the area are 91,040 cattle population, 39,055 goats, 39,048 sheep, 22,676 donkeys, 6,136 horses, and 2,015 mules. However, the total number of pet animals is not yet known [19].

### 2.2. Study Population

The study population was cattle brought to Bishoftu Elfora Abattoir for slaughter. Those animals of all ages and breeds reared under the intensive, semi-intensive, and extensive production and management system were tried to be included in the study for assessing the neoplastic masses on the liver after slaughter.

### 2.3. Study Design

A cross-sectional study design with purposive sampling of animals showing any type of swelling or mass was conducted, and then the neoplastic masses were identified.

## 3. Methods of Sampling and Sample Processing

### 3.1. Gross Examination

A total of 60 liver samples from 60 cattle were taken for assessing HCC from an animal slaughtered at Bishoftu Elfora Abattoir. Out of this, a 10-year-old local bull was found to be affected by a hepatic tumor. On antemortem inspection, the bull exhibited normal clinical parameters (pulse rate, respiratory rate, and temperature) except that the animal was unwilling to move. On postmortem inspection, the liver of the bull revealed multifocal tumorous lesions on different lobes of the liver. Then, the liver was visually and carefully observed and then palpated for consistency, texture, and attachments. Then, the tissue specimens were taken from the liver for further characterization of the lesions.

### 3.2. Histopathology Techniques

The tissue specimens collected for histopathology were immediately fixed in 10% neutral-buffered formalin. Then, the sample was taken to the NAHDIC for sample processing. Formalin-fixed tissues were trimmed and put into plastic tissue cassettes and then processed using an automatic tissue processer. Within the automatic tissue processor, tissues were dehydrated in graded concentrations of absolute alcohol (70%, 95%, and 100%), cleared by xylene, and impregnated/infiltrated by molten paraffin. Impregnated tissues were made into a mold and solidified, and then it was embedded. Tissues were sectioned to 4–5 mm thickness using a semi-automatic microtome machine and then the tissue ribbons were floated on a warm water bath at 45°C, and it became adhered to albumenized glass slides. The sectioned tissues were dewaxed (by heat and xylene), hydrated (by descending grades of alcohol (100%, 95%, and 70%)), and then stained by haematoxylin and eosin (H and E). The stained slides were dehydrated in ascending grades of alcohol (70%, 95%, and 100%), cleared by xylene, then mounted by Canada balsam, and examined under the microscope, and finally, photographs of the slides were taken [20].

### 3.3. Cytologic Sample Collection and Processing

Imprints or impression smears were made from a surgically excised specimen on the slides to appreciate the cellular components at the Addis Ababa University College of Veterinary Medicine in the veterinary pathology laboratory. Any fluid or blood was dabbed off before sampling, and then the slide was placed face down onto the lesion and pressed without rubbing or scraping the slide across the lesion. After the smears were made by this method, it was air-dried, fixed with methanol, and stained with Giemsa. Finally, the stained smears were scanned microscopically starting from lower magnifications to 100x.

### 3.4. Data Analysis

Data generated from field and laboratory investigations were recorded, screened, and coded using Microsoft Excel spreadsheets, and the cytological, gross, and histopathological lesions and findings were described using qualitative methods.

## 4. Results

### 4.1. Gross Characterization of Hepatocellular Carcinomas

**Figure 1:**
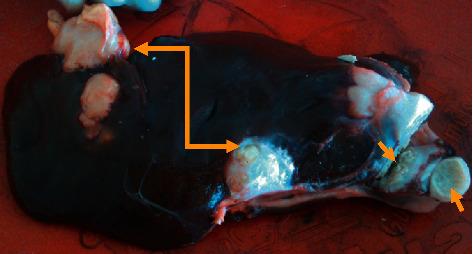
Gross characterization of hepatocellular carcinomas in a bull.

The present study revealed that a bull that was slaughtered at Bishoftu Elfora Abattoir showed a significant gross finding in the liver. Grossly, the liver appeared, hypertrophied with a dull edge, and was characterized by the presence of distinct multiple pale to white foci, soft, spongy nodules (10–40 mm diameter) in different lobes of the liver (Figure 1, double arrow). On the cut surface, tumors had rather sharply circumscribed borders and were divided into lobules by thin connective tissue. The central zone of the tumors exhibited depression with a whitish fibrous area (Figure 1, arrow).

### 4.2. Histopathological Characterization of Hepatocellular Carcinomas

**Figure 2:**
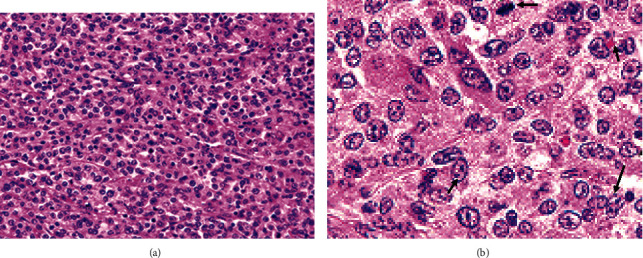
Histopathology of hepatocellular carcinomas of a bull.

Microscopically, the hepatocellular carcinomas were unclearly demarcated without encapsulation and often invading the surrounding hepatic lobules. The tumors were highly cellular and displayed carcinomatous lesions consisting of neoplastic hepatocytes. The tumor cells formed a thick, compact, somewhat ambiguous trabecular pattern of arrangement that was unglued by thin collagenous stroma (Figure 2(a)). Neoplastic hepatocytes exhibited varying-sized nuclei and cytoplasm, eosinophilic granules in the cytoplasm, and mitotic figures (Figure 2(b)). Clear eosinophilic granules in the cytoplasm and the multilobed, giant nucleus are present in tumor cells (Figure 2(b), arrows).

### 4.3. Cytological Characterization of Hepatocellular Carcinomas

**Figure 3:**
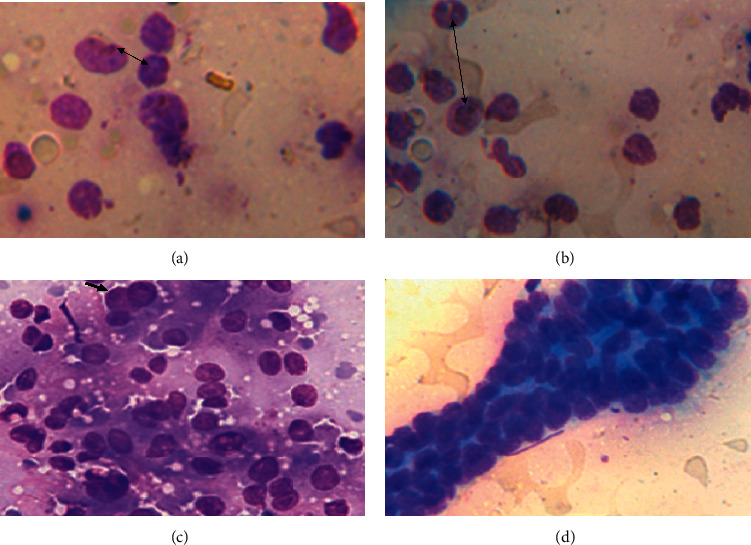
Cytological features of hepatocellular carcinomas in a bull.

Cytologically, hepatocellular carcinomas were characterized by the presence of pleomorphic hepatocytes with nuclear or cytoplasmic variations with an increased nuclear‐to‐cytoplasmic ratio, round or, rarely, spindle shape cells (Figures 3(c) and 3(d)), individual hepatocyte giant cells with enlarged or multilobulated nuclei or multinucleated giant cells (Figure 3(c), arrows), prominent nucleoli, and mitotic figures (Figures 3(a) and 3(b), double-headed arrows). Note the palisading arrangement fusiform cells with eosinophilic cytoplasm and oval nuclei among the small clusters of neoplastic cells (Figure 3(d)).

## 5. Discussion

Tumors have been gaining relevance in veterinary medicine and represent a continuous challenge for veterinarians [21]. Domestic animals may serve as useful environmental sentinels of cancer risks and add further value to the study of naturally occurring tumors in domestic animals which can provide a useful (sometimes irreplaceable) contribution in comparative oncology [22–24]. It is always difficult to estimate the true frequency as well as the biological pattern and clinical outcome of spontaneous tumors in domestic animals [21] even though numerous surveys were conducted in countries throughout the world [5]. The present study reports spontaneously occurring hepatic tumors in a bull. In this study, hepatocellular carcinoma appeared as a distinct grayish-white multifocal soft, friable nodule (10–40 mm in diameter) in different lobes of the liver. The cut surface of the tumors had revealed a sharply circumscribed border, and they were divided into lobules by thin connective tissue. The central zone of the tumors exhibited depression with a whitish fibrous area. The gross features of HCC in the present study concurred with reports of HCC in a cow [2] and a sheep [14]. However, this finding disagreed with [6, 17, 25] which reported that HCC appeared as a massive, unifocal, or diffusely infiltrative mass. Hemorrhage and necrosis were not appreciated in the current study which agreed with the finding of Meuten [3] who reported that focal, usually central, areas of dark red discoloration caused by hemorrhage and necrosis are common in larger tumors which were uncommon in other liver nodules of hepatocellular origin.

The histological appearance of hepatocellular carcinomas varies considerably, depending on the degree of differentiation of the individual hepatocytes and the histological arrangement of the cells [25–27]. In the present study, HCC was microscopically characterized by infiltrative growth, regional or diffuse pleomorphism, and the presence of atypical cells with eosinophilic cytoplasm, nests, or cluster cells embedded in the fibrous stroma, large oval nuclei, and nucleoli or mitotic figure. These histological features corresponded with the findings of [1, 2, 14]. Hepatocellular carcinomas often contain more than one histologic pattern [8]. However, the trabecular pattern is the most common histological form of the tumor in domestic animals reported by [16, 26, 27] and was also observed in the present study.

Cytologically, hepatocellular carcinomas were also diagnosed by its marked cellular atypia including increased N : C ratios, multiple prominent nucleoli, anisocytosis, anisokaryosis, multinuclearity, multiple prominent nucleoli, irregular chromatin patterns, and nuclear outlines, and high mitotic rate was as previously described in [28–32] and concurred with the present findings of HCC in a bull. In the present study, the tumor cells exhibited palisading arrangements of neoplastic cells as reported in [2, 30] Cytoplasmic and intranuclear vacuoles were observed in the current study which was also reported previously by Murakata et al. [33] in humans and Masserdotti and Drigo [34] in dogs. This is due to the presence of abundant lipid or glycogen in the cytoplasm that made it appear as clear. Cytoplasmic basophilia and accumulation of materials such as lipofuscin, glycogen-type material, or lipid vacuoles observed in neoplastic cells in this study were not discriminating features but rather seemed to be a manifestation of metabolic errors in neoplastic cells [35].

## 6. Conclusion and Recommendations

In this study, hepatocellular carcinomas were one of the frequent hepatic neoplasms of cattle characterized at Bishoftu Elfora Abattoir. Grossly, it was characterized by a white to grayish multifocal soft nodule in different lobes of the liver. Histologically, the commonest trabecular pattern was appreciated with different cellular features including anisocytosis, anisokaryosis, increased N : C ratios, multinuclearity, prominent nucleoli, and light eosinophilic cytoplasm. Thus, studies using a large sample size with wide-area coverage and immunohistochemistry should be recommended to have complete data on hepatocellular carcinomas.

## Data Availability

The data used to support the findings of this study are available from the corresponding author upon request.

## Abbreviations

HCC: Hepatocellular carcinoma
NAHDIC: The National Animal Health Diagnostic and Investigation Center
N : C: Nuclear-to-cytoplasmic ratios.

## References

[1] Jeong W. I., Do S. H., Sohn M. H. (2005). Hepatocellular carcinoma with metastasis to the spleen in a Holstein cow. *Veterinary Pathology*.

[2] Ohfuji S. (2016). Hepatocellular carcinoma in a cow: detection of tumor-infiltrating T lymphocytes implicated in anti-tumor immune response and partial spontaneous tumor regression. *Comparative Clinical Pathology*.

[3] Meuten D. J. (2016). *Tumors in Domestic Animals*.

[4] Monlux A., Anderson W., Davis C. (1956). A survey of tumors occurring in cattle, sheep, and swine. *American Journal of Veterinary Research*.

[5] Bastianello S. S. (1982). A Survey on Neoplasia in Domestic Species over a 40-year Period from 1935 to 1974 in the Republic of South Africa. I. Tumours Occurring in Cattle. *Onderstepoort Journal of Veterinary Research*.

[6] Bettini G., Marcato P. S. (1992). Primary hepatic tumours in cattle. A classification of 66 cases. *Journal of Comparative Pathology*.

[7] Haddad J. L., Habecker P. L. (2012). Hepatocellular carcinomas in Vietnamese pot-bellied pigs (Sus scrofa). *Journal of Veterinary Diagnostic Investigation*.

[8] van Sprundel R. G., van den Ingh T. S., Desmet V. J. (2010). Keratin 19 marks poor differentiation and a more aggressive behaviour in canine and human hepatocellular tumours. *Comparative Hepatology*.

[9] de Lope C. R., Tremosini S., Forner A., Reig M., Bruix J. (2012). Management of HCC. *Journal of Hepatology*.

[10] Lin S., Hoffmann K., Schemmer P. (2012). Treatment of hepatocellular carcinoma: a systematic review. *Liver Cancer*.

[11] Kato H., Nakamura M., Muramatsu M., Orito E., Ueda R., Mizokami M. (2004). Spontaneous regression of hepatocellular carcinoma: two case reports and a literature review. *Hepatology Research*.

[12] Meza-Junco J., Montaño-Loza A. J., Martinez-Benítez B., Cabrera-Aleksandrova T. (2007). Spontaneous partial regression of hepatocellular carcinoma in a cirrhotic patient. *Annals of Hepatology*.

[13] Huz J. I., Melis M., Sarpel U. (2012). Spontaneous regression of hepatocellular carcinoma is most often associated with tumour hypoxia or a systemic inflammatory response. *Hpb*.

[14] Gholami M., Habl A. M., Ezi A. (2006). *Hepatocellular Carcinoma in Sheep*.

[15] Liptak J. M., Dernell W. S., Monnet E. (2004). Massive hepatocellular carcinoma in dogs: 48 cases (1992-2002). *Journal of the American Veterinary Medical Association*.

[16] Cullen J. M. (2016). Tumors of the liver and gallbladder. *Tumors in Domestic Animals*.

[17] Szklaruk J., Silverman P. M., Charnsangavej C. (2003). Imaging in the diagnosis, staging, treatment, and surveillance of hepatocellular carcinoma. *American Journal of Roentgenology*.

[18] Belay H., Muktar Y. (2015). Isolation and identification of foot and mouth disease virus from clinically infected cattle in ada veterinary clinic. *American Eurasian Journal of Scientific Research*.

[19] Genzebu D., Tamir B., Berhane G. (2016). Study of reproductive and production performance of cross breed dairy cattle under smallholders management system in Bishoftu and Akaki towns. *International Journal of Advanced Research in Biological Sciences*.

[20] Talukder S. (2007). *Histopathology techniques: tissue processing and staining*.

[21] D’Angelo A. R., Vita S., Marruchella G., Di Francesco G. (2012). Canine testicular tumours: a retrospective investigation in Abruzzo and Molise, Italy. *Veterinaria Italiana*.

[22] Hayes H. M., Tarone R. E., Casey H. W., Huxsoll D. L. (1990). Excess of seminomas observed in vietnam service U.S. Military working dogs. *JNCI: Journal of the National Cancer Institute*.

[23] Reif J. S. (2011). Animal sentinels for environmental and public health. *Public Health Reports*.

[24] Pinho S. S., Carvalho S., Cabral J., Reis C. A., Gärtner F. (2012). Canine tumors: a spontaneous animal model of human carcinogenesis. *Translational Research*.

[25] Trigo F. J., Thompson H., Breeze R. G., Nash A. S. (1982). The pathology of liver tumours in the dog. *Journal of Comparative Pathology*.

[26] Rooney J. R. (1959). Liver carcinoma in the dog. *Acta Pathol Microbiol Scand*.

[27] Patnaik A. K., Hurvitz A. I., Lieberman P. H., Johnson G. F. (1981). Canine hepatocellular carcinoma. *Veterinary Pathology*.

[28] Wee A., Nilsson B., Tan L., Yap I. (1994). Fine needle aspiration biopsy of hepatocellular carcinoma. Diagnostic dilemma at the ends of the spectrum. *Acta Cytologica*.

[29] Pedio G., Landolt U., Zöbeli L., Gut D. (1988). Fine needle aspiration of the liver. Significance of hepatocytic naked nuclei in the diagnosis of hepatocellular carcinoma. *Acta Cytologica*.

[30] Cohen M. B., Haber M. M., Holly E. A., Ahn D. K., Bottles K., Stoloff A. C. (1991). Cytologic criteria to distinguish hepatocellular carcinoma from nonneoplastic liver. *American Journal of Clinical Pathology*.

[31] Roth L. (2001). Comparison of liver cytology and biopsy diagnoses in dogs and cats: 56 cases. *Veterinary Clinical Pathology*.

[32] Stockhaus C., Teske E., Van Den Ingh T., Rothuizen J. (2002). The influence of age on the cytology of the liver in healthy dogs. *Veterinary Pathology*.

[33] Murakata L. A., Ishak K. G., Nzeako U. C. (2000). Clear cell carcinoma of the liver: a comparative immunohistochemical study with renal clear cell carcinoma. *Modern Pathology*.

[34] Masserdotti C., Drigo M. (2012). Retrospective study of cytologic features of well-differentiated hepatocellular carcinoma in dogs. *Veterinary Clinical Pathology*.

[35] Ishak KG G. Z., Stocker J. T. (2001). *Tumors of the liver and intrahepatic bile ducts (AFIP)*.

